# Characterising pharmacists’ interventions in chronic non-cancer pain care: a scoping review

**DOI:** 10.1007/s11096-024-01741-x

**Published:** 2024-06-11

**Authors:** Aljoscha Noël Goetschi, Carla Meyer-Massetti

**Affiliations:** 1 Clinical Pharmacology and Toxicology, General Internal Medicine, University Hospital of Bern, Anna-Von-Krauchthal-Weg 7, 3010 Bern, Switzerland; 2 Graduate School for Health Sciences, University of Bern, Bern, Switzerland; 3 Institute of Primary Health Care (BIHAM), University of Bern, Bern, Switzerland

**Keywords:** Clinical pharmacy, Chronic pain, Medication safety, Pharmaceutical services, Pain management, Pharmacist

## Abstract

**Background:**

Chronic non-cancer pain may affect up to 51% of the general population. Pharmacist interventions have shown promise in enhancing patient safety and outcomes. However, our understanding of the scope of pharmacists’ interventions remains incomplete.

**Aim:**

Our goal was to characterise pharmacists’ interventions for the management of chronic non-cancer pain.

**Method:**

Medline, Embase, PsycINFO via Ovid, CINAHL via EBSCO databases and the Cochrane Library were systematically searched. Abstracts and full texts were independently screened by two reviewers. Data were extracted by one reviewer, and validated by the second. Outcomes of studies were charted using the dimensions of the Initiative on Methods, Measurement, and Pain Assessment in Clinical Trials (IMMPACT).

**Results:**

Forty-eight reports were included. Interventions ensuring appropriate drug prescription occurred in 37 (79%) studies. Patient education and healthcare professional education were reported in 28 (60%) and 5 (11%) studies, respectively. Therapy monitoring occurred in 17 (36%) studies. Interventions regularly involved interprofessional collaboration. A median of 75% of reported outcome domains improved due to pharmacist interventions, especially patient disposition (adherence), medication safety and satisfaction with therapy.

**Conclusion:**

Pharmacists’ interventions enhanced the management of chronic non-cancer pain. Underreported outcome domains and interventions, such as medication management, merit further investigation.

**Supplementary Information:**

The online version contains supplementary material available at 10.1007/s11096-024-01741-x.

## Impact statements

- Pharmacists are well-positioned to improve the management of chronic non-cancer pain.
- Pharmacists should consider multimodal, interprofessional interventions for the management of chronic non-cancer pain.
- Future opportunities for pharmacists lie in holistic, patient-centred approaches such as medication management.

## Introduction

Chronic non-cancer pain (CNCP) affects a substantial portion of the population, with at least one-fifth of individuals in the US and Europe experiencing this condition at one point during their lives (19–51%) [1, 2]. CNCP induces a significant burden on overall quality of life, often including depression and insomnia [3–6]. CNCP is more common among women [7], older adults [8] and people from poor socioeconomic background [9]. As defined by the International Association for the Study of Pain, chronic pain, including CNCP, lasts for over three months [10]. There are various causes of CNCP, often necessitating effective multimodal management strategies, combining physical, psychological and pharmacological therapies [11].

Pharmacists play crucial roles in enhancing medication safety in a variety of settings and patient populations, offering services such as prescription validation, patient counselling and therapeutic drug monitoring [12]. Their involvement in medication reviews (MRs) and dosage adjustments contribute to appropriate CNCP management [13]. Indeed, positive effects have been found by three preceding systematic reviews regarding pharmacists’ interventions in CNCP management: a 2011 systematic review and meta-analysis found that patient education by pharmacists reduced pain intensity [14]; a 2014 systematic review exploring pharmacists performing MRs also found lower levels of pain [15]; and a more recent systematic review and meta-analysis reported similar effects for interventions not limited to education or MRs [16]. Altogether, current evidence suggests that pharmacists enhance the quality of care provided to patients with CNCP.

However, these three reviews only provided limited insight into the type and structure of these interventions [14–16]. When pharmacists seek to contribute to CNCP care, they often lack a comprehensive understanding of which interventions they could implement in this context. This scoping review is intended to bridge this information gap.

### Aim

The aim of this scoping review was to concisely yet comprehensively identify, describe and categorise the current range and attributes of pharmacists’ interventions and services for managing patients with CNCP. The secondary objective was to descriptively synthesise reported outcomes.

## Method

### Protocol and design

Prior to starting this review, a protocol was published [17], and the Preferred Reporting Items for Systematic Reviews and Meta-Analyses’ extension for scoping reviews (PRISMA-Scr) [18] was followed in reporting.

### Eligibility criteria

The studies included involved adult patients (≥ 18 years) suffering from CNCP. Author-defined CNCP or pain described as lasting more than three months were both accepted. Patients with cancer-related pain and patients addicted to opioids were excluded. All pharmacist interventions aimed at patients with CNCP were included, except for interventions solely targeting opioid addiction or opioid tapering. If the intervention included opioid tapering but also focused on improving CNCP care (i.e. by providing patient education), the study was included. Interventions delivered in any healthcare setting were accepted, as were trials with or without controls. Primary literature with any study design was included (except case studies). Non-peer-reviewed papers, conference abstracts, conference proceedings, editorials, commentaries and literature reviews were excluded. There were no restrictions on dates or the language of publication.

### Information sources

Sources of evidence were retrieved from Medline, Embase and PsycINFO databases via Ovid, as well as from the CINAHL database via EBSCO and the Cochrane Library. Additionally, backward citation chasing was performed on the studies retrieved using the Citationchaser tool [19]. The first 100 citations, sorted by relevance, were screened using Google Scholar.

### Search

Ovid was used to create the search string for the Medline database, and the search string was validated using articles from previous systematic reviews [14–16] that met the inclusion criteria as seed papers and in collaboration with the University of Basel’s Medical Library. The search strategy was translated for other databases using the Systematic Review Accelerator® [20]. The search consisted of two thematic search blocks, ‘pharmacists’ and ‘chronic non-cancer pain’. Each block combined MeSH or Emtree terms as well as free text searches limited to title and abstract. The search strings are shown in Supplementary Information 1. The search was conducted on 12 October 2023.

### Selecting sources of evidence

The selection processes were carried out using Covidence® systematic review software (Veritas Health Innovation, Melbourne, Australia.). Duplicate articles were also removed with Covidence®. Titles and abstracts were independently screened by both authors based on the inclusion and exclusion criteria. The full texts of the studies retained were retrieved. The full-text information was independently compared with the inclusion and exclusion criteria by both authors, and a final decision was made. In cases of disagreement, the authors again resolved them through discussion.

### Data items

Pre-defined data items were retrieved as per the review protocol [17]. General study details and methodological information were tabulated. Information about the pharmacist interventions (types and characteristics) was extracted. Aims, outcomes, results and conclusions were also included.

### Data charting

Data were charted using the three dimensions of intervention type, setting and outcomes. Intervention types were charted using an adapted framework based on the definition of clinical pharmacy published in a European Society of Clinical Pharmacy policy paper [21] (Table 1). It is worth noting that, each intervention could combine multiple intervention types. Studies were also charted according to their healthcare setting, distinguishing between acute care (e.g. hospitals), ambulatory care (e.g. outpatient clinics in hospitals) and primary care (e.g. primary care centres, community pharmacies). Furthermore, various outcomes reported by the study authors were charted following an adapted methodology used by Gondora et al*.* [22] and using the core outcome domains for chronic pain clinical trials in the Initiative on Methods, Measurement, and Pain Assessment in Clinical Trials’ (IMMPACT) guidelines [23]. The IMMPACT domains encompass: (1) pain intensity, (2) physical function, (3) emotional well-being, (4) participant ratings of satisfaction or improvement with therapy, (5) symptoms and ADEs, and (6) participant disposition (e.g. adherence to the therapy). An evaluation of costs was added as a seventh domain. The number of domains each study covered and whether they improved, stayed the same or deteriorated was assessed. When a domain improved, one point was awarded; when it deteriorated, one point was subtracted; and when no change occurred in the domain, no points were awarded. A half point was added or subtracted for partial improvements or partial deteriorations. A domain was considered to have partially improved or deteriorated if not all reported variables for that domain showed improvement or deterioration. Each study’s ratio of points awarded per outcome domain was calculated. The median improvement in each domain and the total number of times each domain was mentioned were reported. These two numbers made it possible to compute the median percentage of improved domains. To depict the distribution of medians, their inter-quartile range (IQR) was reported. In addition, the reported outcome domains were compared for different types of intervention. Using those categories, data charting was completed by the first reviewer (AG) and verified by the second (CMM). In cases of disagreement, the reviewers sought consensus through discussion.

**Table 1:** The different categories used for data charting on pharmacist interventions. Categories were taken from the European Society of Clinical Pharmacy’s (ESCP) definition of clinical pharmacy [21]. We further specified those categories and gave concise definitions

Adapted ESCP category [21]	Specification	Definition
Drug prescription	Medication reconciliation	Activities to ensure the most complete and accurate medication history
	Medication review	Structured analysis of medication regimens to inform appropriate drug initiation, switching or deprescribing Sub-Category: remote versus with patient contact
	Medication management	Independent management of patients’ medications involving initiating, switching or stopping therapies (including non-pharmacological options)
Education	Patient education	Patient education regarding diseases or prescriptions, but also teaching coping strategies or increasing adherence
	Healthcare professional education	Healthcare professional education, including nurses and physicians
Drug monitoring	Monitoring	Monitoring current therapies, detecting adverse events or non-adherence, and handling the resulting problems
Compounding	Compounding	Extemporaneous production of medications used to treat CNCP

### Synthesis of results

The results were analysed and described in a narrative synthesis, and the results were categorised to assess the frequency of intervention types.

## Results

### Selection of sources of evidence

**Fig. 1:**
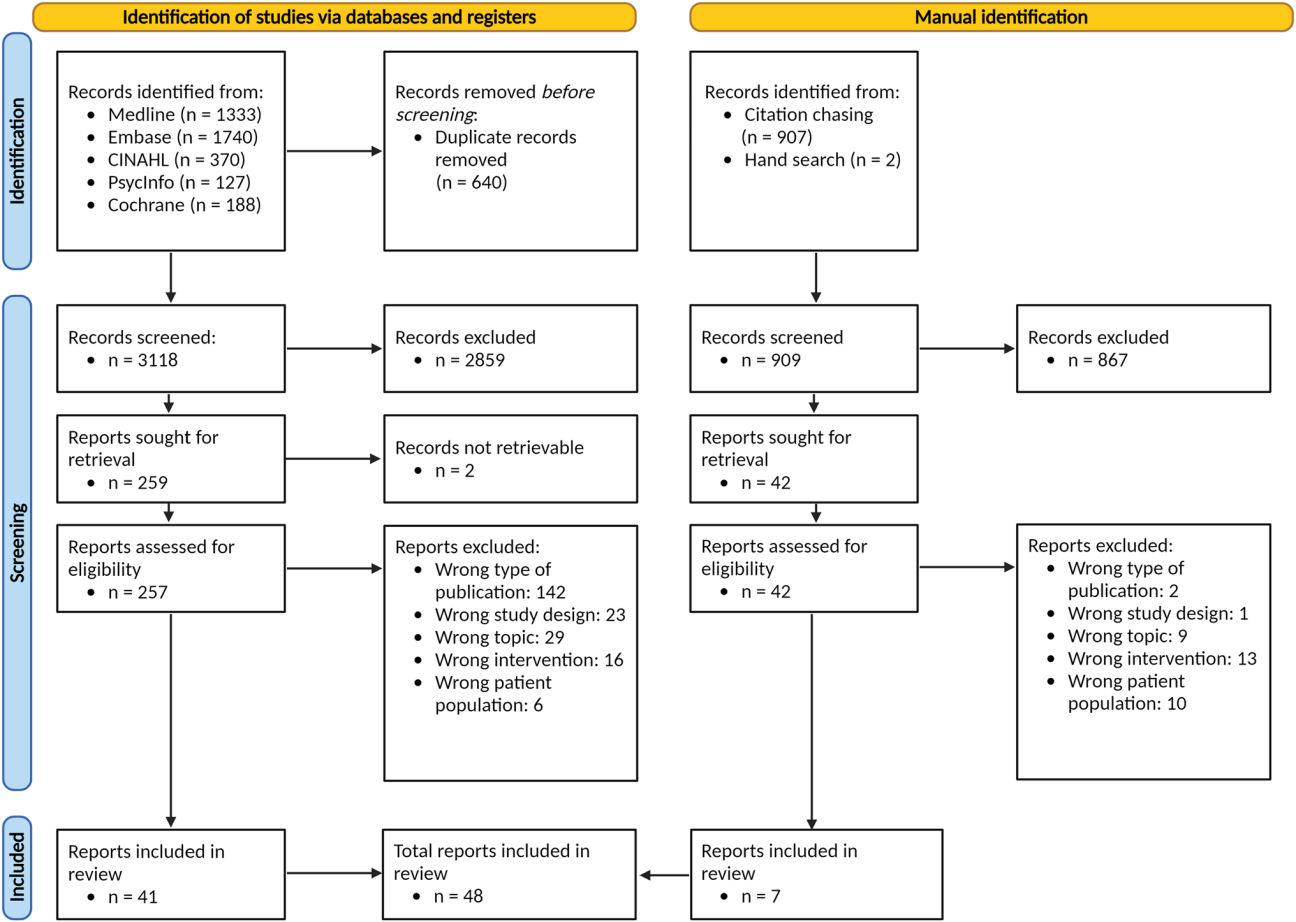
PRISMA-Scr flow diagram [18]. Created with BioRender.com

The combined search found 3758 published articles across the five databases. Based on our defined criteria, 48 reports were included [24–71]. One study report [59] was a secondary analysis of another [32], so those papers’ insights were combined. Among these 47 different interventional studies, 41 were identified through the systematic search and 7 through citation chasing and hand searching. Figure 1 depicts the study’s PRISMA flow diagram [18].

### Characteristics of sources of evidence

The studies included had varied characteristics, with 29 (62%) originating from North America, 13 (38%) from Europe, three (6%) from Australia, and two (4%) from Asia. Sixteen (34%) studies were pre–post studies, 11 (23%) were cross-sectional studies, six (13%) were randomised controlled trials, four (9%) were chart reviews and 10 (21%) employed other study designs.

Patients with any form of CNCP were included in the majority of studies (*n* = 42, 90%), although some interventions targeted specific subpopulations. Two (4%) interventions were directed at patients with arthritis, two (4%) targeted patients with migraine, and one (2%) was aimed specifically at patients with neuropathic pain.

### Results of individual sources of evidence

The information and charted interventions extracted from each article included are presented in Table 2.

**Table 2:** Charted intervention types, the scope of practice of the pharmacists involved and the other professions involved per study

	Sum (%)	Alibaud R. et al. [24]	Barrachina J. et al. [25]	Bauters T. et al. [26]	Bellnier T. et al. [27]	Bhimji H. et al. [28]	Boren L. et al. [29]	Briggs M. et al. [30]	Bright D. et al. [31]	Bruhn H. et al. [32]^a^	Chelminski P. et al. 33]	Chen M. et al. [34]	Coffey C. et al. [35]	Conley M. et al. [36]	Cox N. et al. [37]	Dawson K. et al. [38]
*Interventions*																
Drug prescription	37 (79)	X		X	X	X	X	X	X	XY	X	X	X		X	X
MedRec	5 (11)	X												X		X
Remote MR	14 (30)			X					X	X					X	
FtF MR	20 (43)					X	X	X		Y		X	X			X
Medication management	11 (23)				X		X			Y	X					
Education	31 (66)	X		X	X	X						X	X			X
Healthcare professional education	5 (11)			X												
Patient Education	28 (60)	X			X	X				y		X	X			X
Monitoring	17 (36)		X		X		X			Y	X	X	X			
Compounding	1 (2)															
*Scope of practice*																
Prescription rights	9 (19)				X		X	X		Y						
Advising physicians/patients	38 (81)	X	X	X		X			X	X	X	X	X	X	X	X
*Professions*																
Medical doctors	42 (89)	X	X	X	X	X	X	X	X	X	X	X	X	X	X	X
Nurses	14 (30)	X	X	X				X						X		
Psychologists	4 (9)			X							X					
Physiotherapists	6 (13)															
Occupational therapists	2 (4)		X													
Social workers	2 (4)			X												
Behavioural therapy	1 (2)															
Dieticians	2 (4)															
Biologists	1 (2)		X													

		DeBar L. et al. [39]		Dole E. et al. [40]		Duvivier H. et al. 41]		Faley B. et al. 42]		Fong [43]		Gammaitoni A. et al. 44]		Hadi M. et al. 45]		Hay E. et al. [46]		Hoffmann W. et al. 47]		Jorgenson D. et al. 48]		Joypaul S. et al. 49]		Keen A. et al. [50]		Kientz J. et al. [51]		Kroner B. et al. [52]			Lagisetty P. et al. [53]		Maleki S. et al. [54]
*Interventions*																																	
Drug prescription		X		X		X		X				X		X		X		X		X								X			X		X
MedRec																										X							X
Remote MR		X						X												X								X					X
FtF MR						X		X				X		X		X		X													X		
Medication management				X												X		X		X													
Education				X		X		X		X		X		X				X				X		X		X							X
Healthcare professional education										X														X									X
Patient education				X		X		X				X		X				X				X				X							X
Monitoring				X								X				X		X		X													
Compounding										X																							
Scope of practice																																	
Prescription rights				X				X								X																	
Advising physicians/patients		X				X				X		X		X				X		X		X				X		X			X		X
*Professions*																																	
Medical doctors		X		X		X		X		X				X		X		X		X		X				X		X			X		X
Nurses		X		X						X		X		X								X		X		X							
Psychologists										X																							
Physiotherapists		X								X										X		X				X							
Occupational therapists										X																							
Social workers																				X													
Behavioural therapy		X																															
Dieticians																						X											
Biologists																																	

		Manzur V. et al. [55]		Mayfield K. et al. [56]	McDermott et al. [57]	Mínguez Martí et al. [58]		Norman J. et al. [60]	Petkova [61]	Read R. et al. [62]		Richet E. et al. [63]	Rife T. et al. [64]	Semerjian M. et al. [65]		Skomo M. et al. [66]	Slipp M. et al. [67]	Takahashi N. et al. [68]	Tilli T. et al. [69]	Uejima K. [70]	WeidmanEvans E. et al. [71]
*Interventions*																					
Drug prescription		X		X	X	X		X		X		X	X	X			X		X		X
MedRec																					
Remote MR					X							X	X						X		X
FtF MR		X		X				X		X				X			X				
Medication management						X								X			X				
Education		X		X				X	X	X		X		X		X		X	X	X	X
Healthcare professional education																					X
Patient education		X		X				X	X	X		X		X		X		X	X	X	X
Monitoring				X		X		X				X		X							
Compounding																					
Scope of practice																					
Prescription rights														X			X				
Advising physicians/patients		X		X	X	X		X	X	X		X	X			X		X	X	X	X
Professions																					
Medical doctors		X		X	X	X		X		X		X	X	X			X	X	X		X
Nurses																		X			
Psychologists																		X			
Physiotherapists																		X			
Occupational therapists																					
Social workers																					
Behavioural therapy																					
Dieticians																		X			
Biologists																					

^a^ This study analysed two different interventions. The first is denoted with an X, and the second with a Y

* MedRec* medication reconciliation,* Remote MR* remote medication review,* FtF MR* face-to-face medication review

### Synthesis of results

The included studies described interventions in different settings. Ambulatory care settings were reported on in 21(45%) studies, primary care settings in 18 (38%) and acute care settings in 6 (13%), with 1 (2%) in a university (2%) and 1 (2%) in a prison service facility.

Intervention characteristics varied across the included studies. Multi-component interventions were present in 30 (64%) studies, and single-component interventions were presented in 17 (36%). Drug prescription interventions were presented in 37 (79%) studies, of which 20 (43%) reported face-to-face MRs, 14 (30%) reported remote MRs, 11 (23%) reported medication management and 5 (11%) reported medication reconciliation. Educational interventions were reported in 31 (66%) studies, with 5 (11%) describing educational interventions involving healthcare professionals (HPs) and 28 (60%) describing them with patients. Monitoring interventions were described in 17 (36%) studies, and compounding was reported in one (2%).

Pharmacists frequently cooperated with other HPs when providing care for patients with CNCP. Physicians were involved in 42 (89%) studies, nurses in 14 (30%) and physiotherapists in 6 (13%). Other less frequently involved professions included psychologists (*n* = 4, 9%), dieticians (*n* = 2, 4%), social workers (*n* = 2, 4%), occupational therapists (*n* = 2, 4%), behavioural therapists (*n* = 1, 2%) and biologists (*n* = 1, 2%).

The scope of pharmacists’ practice differed across the reported interventions. In 9 (19%) studies, pharmacists possessed prescribing rights and managed their patients independently; in the 38 (81%) remaining studies, pharmacists either recommended medication therapy optimisations to physicians and/or advised patients directly.

The studies reported a median of two outcome domains (interquartile range (IQR): 1–4) and, of these, a median of 1.5 domains improved (IQR: 1–2), corresponding to a median of 75% improved outcomes per study. Forty studies reported on symptoms and ADEs, 24 on pain intensity, 22 on physical function, 18 on emotional well-being, 14 on satisfaction with therapy, 9 on patient disposition and 6 on the costs of the intervention. Except for physical function, all the domains had positive outcomes in the majority of studies. The highest number of positive outcomes were reported in the domain of adherence (8/9 studies were positive, 89%), followed by symptoms and ADEs (34/40 positive, 85%), satisfaction with therapy (11.5/14 positive, 82%), the costs of the intervention (4/6 positive, 67%), pain intensity (15/24 positive, 63%), emotional well-being (10/18 positive, 56%) and physical function (9/22 positive, 41%). More information on the respective intervention types is shown in Table 3 and Supplementary Information 2.

**Table 3:** Summary of the median reported and improved outcome domains, and their ratio

Intervention type		Median improved domains (IQR)	Median reported domains (IQR)	Ratio of improved domains (%)
Drug prescription		1.5 (1–2)	2 (2–4)	75
	Medication reconciliation	1 (1–4)	1 (1–4)	100
	Remote MR	1 (1–2)	2 (2–4)	50
	Face-to-face MR	2 (1–3)	4 (2–5)	50
	Medication management	2 (1–2)	2 (1–5)	100
Education		2 (1–3)	2.5 (1–4)	80
	Patient education	2 (1–3)	3 (1–5)	67
	HP education	1 (1–5)	1 (1–1.5)	100
Monitoring		1.5 (1–2)	3 (1–4)	50
All interventions		1.5 (1–2)	2 (1–4)	75
	Singe-component	1 (1–2.5)	2 (1–4)	50
	Multi-component	2 (1–2)	2 (1–4.5)	100

Summary measures were categorised by reported intervention type

*IQR* inter-quartile range, *MR* medication review, *HP education* healthcare professional education

## Discussion

### Statement of key findings

This scoping review included 47 studies and identified a diverse range of pharmacist interventions aimed at improving the care of patients with CNCP. These interventions took place in a variety of healthcare settings, with most occurring in ambulatory care, followed by primary and then acute care. The most frequently reported interventions focused on drug prescriptions. Among these, face-to-face MRs were the most common, followed by remote MRs, medication management and medication reconciliation. Studies often combined multiple intervention types. Out of seven possible outcome domains—pain intensity, physical functioning, psychological well-being, patient satisfaction, symptoms and ADEs, patient disposition, and costs—study authors reported a median of two domains, of which a median of 1.5 domains of intervention showed positive outcomes.

### Strengths and weaknesses

This review has some limitations. Although our search strategy comprised some diseases closely associated with chronic pain (e.g. migraine), it was not exhaustive in terms of individual conditions that may be associated with chronic pain. Therefore, some studies reporting pharmacist interventions targeting other specific diseases (e.g. arthritis), but not specifically mentioning chronic pain (or a synonym thereof), may have been missed. Full-text data were extracted by one reviewer alone, and a second reviewer verified the retrieved information, so this may have introduced bias. With few studies originating from Asia, and none from Africa or South America, this may limit the generalisability of the results of this review.

The main strength of this scoping review is the extensive search over five large databases, including a citation chase and an extensive hand search. Another strength is the presentation of reported outcomes on the IMMPACT domains, which represent the relevant aspects of CNCP care.

### Interpretation

In this scoping review, a median of 75% of reported outcome domains improved thanks to pharmacist interventions, aligning with three existing systematic reviews that consistently reported the positive effects of pharmacist interventions for CNCP management [14–16]. Bennet et al*.* reported on pain intensity, symptoms and ADEs, emotional well-being and satisfaction with therapy [14]; Hadi et al*.* reported on pain intensity, physical function and satisfaction with treatment [15]; and Thapa et al*.* reported on pain intensity, physical function, emotional well-being, satisfaction with therapy and costs [16]. To the best of our knowledge, there have been no systematic reviews covering all of the outcome domains that should be considered according to the IMMPACT guidelines [23]. Improvements in pain intensity and satisfaction with therapy outcomes were reported by all three reviews, aligning with this review’s qualitative synthesis of author-reported outcomes.

The outcome domains of symptoms and ADE (85%) and of patient disposition (89%) in the included studies had high percentages of improved outcomes. Bennet et al*.* found a significant reduction of ADEs in the interventions reviewed [14]. However, none of the existing systematic reviews considered adherence. Pharmacists are often seen as medication experts, and there is evidence in studies on other diseases that pharmacists can enhance adherence [72]. This suggests that the evidence of the benefits of pharmacist interventions on symptoms and ADEs and on patient disposition, reported in this scoping review, are plausible.

The predominance and greater impact of interventions including some form of MR or patient education found in this review align with previous research in this area. Existing systematic reviews have explored the impact of pharmacist interventions in CNCP management, with some focusing specifically on patient education [14] or MRs [15], indicating the importance of these intervention types. A third systematic review, that did not restrict intervention types, identified 8 out of 14 studies as MR interventions [16], which explains why MR has been implemented repeatedly. Interestingly, in this scoping review, remote and face-to-face MR showed the same median improvement. However, remote MR interventions reported fewer outcome domains than face-to-face MR. This emphasises the potentially more comprehensive approach of a face-to-face MR that allows for patient involvement. The highest median improvements in outcomes were shown by interventions including medication reconciliation, HP education and medication management. However, these three intervention types only comprised a few reports each, making generalisation more difficult. Medication management, in particular, is promising because it involves the pharmacist holistically in CNCP care. It also requires continuous follow-up and patient contact, both of which are known to contribute to improved patient outcomes [11, 73].

In the included studies, pharmacists mostly took a multimodal approach by combining diverse interventions (e.g. face-to-face MR and patient education) and collaborated with other professionals, mainly physicians and nurses, to deliver their interventions, which is widely recommended [11, 73]. This recommendation is supported by this scoping review’s finding that multi-component interventions reported more improved outcomes than single-component interventions.

### Further research

Further research into pharmacist interventions in CNCP care should prioritise less-researched interventions, such as medication management, alongside investigating less conventional roles for pharmacists, such as independent prescribing. Emphasis should be placed on direct patient contact, adequate follow-up and a multimodal approach. In addition, to facilitate the development of rational and effective interventions, more research is needed to determine which types of pharmacist interventions have the highest impact on reported outcomes. Comprehensive reporting should include all the relevant IMMPACT domains, particularly the effect of pharmacist interventions on patient disposition (adherence), patient satisfaction and costs.

## Conclusion

This scoping review revealed a diverse landscape of pharmacist interventions targeting patients with CNCP. Intervention types that addressed appropriate drug prescribing, educated healthcare professionals or patients, monitored treatments and offered compounding were identified. Pharmacist activities often combined multiple intervention types via interprofessional teams. A median of 75% of outcomes in all the reported outcome domains improved after the implementation of pharmacist interventions. However, the effects of pharmacist interventions on important outcome domains, such as costs, patient disposition and satisfaction with the therapy, and their role in interventions such as medication management, remain underreported and require further study.

## Supplementary Information

Below is the link to the electronic supplementary material.

Supplementary file1 (DOCX 19 KB)

Supplementary file2 (DOCX 77 KB)
