# Supplementary material for: Characterising pharmacists’ interventions in chronic non-cancer pain care: a scoping review
Source: Int J Clin Pharm. 2024 Jun 11;46(5):1010–23. doi: 10.1007/s11096-024-01741-x (PMC11399199; doi:10.1007/s11096-024-01741-x)
Supplement: Supplementary file 1 — Supplementary file1 (DOCX 19 KB) [file 11096_2024_1741_MOESM1_ESM.docx]

# Supplementary information 1

**Characterising Pharmacists’ Interventions in Chronic Non-Cancer Pain Care: A Scoping Review**

**International Journal of Clinical Pharmacy**

Aljoscha Goetschi^a,b,*^, Carla Meyer-Massetti^a,c^

^a^Clinical Pharmacology and Toxicology, General Internal Medicine, University Hospital of Bern, Bern, Switzerland

^b^Graduate School of Health Sciences, University of Bern, Bern, Switzerland

^c^Institute for Primary Health Care (BIHAM), University of Bern, Bern, Switzerland

*corresponding author: aljoscha.goetschi@insel.ch

**Search Pubmed via Ovid**

| **#** | **String** | **Results**  **(October 12, 2023)** |
| --- | --- | --- |
| 1 | exp chronic pain/ or ((chronic or persistent or relentless or enduring or constant or sustained or continuous or lingering or protracted or life-long or continual or continuing or recurrent or recurring) adj4 (pain or ache* or cramp* or spasm* or colic* or sore* or tender* or burn* or arthralgia or headache or earache or migraine or mastodynia or allodynia or neuralgia or hyperalgesia or '*myalgia')).ti,ab. | 124928 |
| 2 | exp Pharmacists/ or exp Pharmacy Service, Hospital/ or exp Community Pharmacy Services/ or exp Pharmacy/ or exp Evidence-Based Pharmacy Practice/ or exp Pharmaceutical Services/ or (('pharmaceutical' adj2 service$') or 'pharmaceutical care' or pharmacies* or pharmacy* or pharmacist*).ti,ab. | 147924 |
| 3 | 1 AND 2 | 1333 |

**Search Embase via Ovid**

| **#** | **String** | **Results**  **(October 12, 2023)** |
| --- | --- | --- |
| 1 | exp "chronic pain"/ OR ((chronic OR persistent OR relentless OR enduring OR constant OR sustained OR continuous OR lingering OR protracted OR life-long OR continual OR continuing OR recurrent OR recurring ) ADJ4 (pain OR ache* OR cramp* OR spasm* OR colic* OR sore* OR tender* OR burn* OR arthralgia OR headache OR earache OR migraine OR mastodynia OR allodynia OR neuralgia OR hyperalgesia OR '*myalgia' )).ti,ab. | 201116 |
| 2 | exp Pharmacists/ or exp "Pharmacy Service, Hospital"/ or exp "Community Pharmacy Services"/ or exp Pharmacy/ or exp "Evidence-Based Pharmacy Practice"/ or exp "Pharmaceutical Services"/ or (('pharmaceutical' adj2 service$') or "'pharmaceutical care'" or pharmacies* or pharmacy* or pharmacist*).ti,ab. | 211287 |
| 3 | 1 AND 2 | 1740 |

**Search CINAHL via EBSCO**

| **#** | **String** | **Results**  **(October 12, 2023)** |
| --- | --- | --- |
| 1 | (MH "chronic pain+") OR (((TI chronic OR AB chronic) OR (TI persistent OR AB persistent) OR (TI relentless OR AB relentless) OR (TI enduring OR AB enduring) OR (TI constant OR AB constant) OR (TI sustained OR AB sustained) OR (TI continuous OR AB continuous) OR (TI lingering OR AB lingering) OR (TI protracted OR AB protracted) OR (TI life-long OR AB life-long) OR (TI continual OR AB continual) OR (TI continuing OR AB continuing) OR (TI recurrent OR AB recurrent) OR (TI recurring OR AB recurring)) N4 ((TI pain OR AB pain) OR (TI ache* OR AB ache*) OR (TI cramp* OR AB cramp*) OR (TI spasm* OR AB spasm*) OR (TI colic* OR AB colic*) OR (TI sore* OR AB sore*) OR (TI tender* OR AB tender*) OR (TI burn* OR AB burn*) OR (TI arthralgia OR AB arthralgia) OR (TI headache OR AB headache) OR (TI earache OR AB earache) OR (TI migraine OR AB migraine) OR (TI mastodynia OR AB mastodynia) OR (TI allodynia OR AB allodynia) OR (TI neuralgia OR AB neuralgia) OR (TI hyperalgesia OR AB hyperalgesia) OR (TI '*myalgia' OR AB '*myalgia'))) | 63560 |
| 2 | (MH Pharmacists+) OR (MH "Pharmacy Service, Hospital+") OR (MH "Community Pharmacy Services+") OR (MH Pharmacy+) OR (MH "Evidence-Based Pharmacy Practice+") OR (MH "Pharmaceutical Services+") OR (((TI 'pharmaceutical' OR AB 'pharmaceutical') N2 (TI service?' OR AB service?')) OR (TI "'pharmaceutical care'" OR AB "'pharmaceutical care'") OR (TI pharmacies* OR AB pharmacies*) OR (TI pharmacy* OR AB pharmacy*) OR (TI pharmacist* OR AB pharmacist*)) | 49967 |
| 3 | 1 AND 2 | 370 |

**Search PsycInfo via Ovid**

| **#** | **String** | **Results**  **(October 12, 2023)** |
| --- | --- | --- |
| 1 | exp "chronic pain"/ OR ((chronic.ti,ab. OR persistent.ti,ab. OR relentless.ti,ab. OR enduring.ti,ab. OR constant.ti,ab. OR sustained.ti,ab. OR continuous.ti,ab. OR lingering.ti,ab. OR protracted.ti,ab. OR life-long.ti,ab. OR continual.ti,ab. OR continuing.ti,ab. OR recurrent.ti,ab. OR recurring.ti,ab.) ADJ4 (pain.ti,ab. OR ache*.ti,ab. OR cramp*.ti,ab. OR spasm*.ti,ab. OR colic*.ti,ab. OR sore*.ti,ab. OR tender*.ti,ab. OR burn*.ti,ab. OR arthralgia.ti,ab. OR headache.ti,ab. OR earache.ti,ab. OR migraine.ti,ab. OR mastodynia.ti,ab. OR allodynia.ti,ab. OR neuralgia.ti,ab. OR hyperalgesia.ti,ab. OR '*myalgia'.ti,ab.)) | 32011 |
| 2 | exp Pharmacists/ or exp Pharmacy/ or (('pharmaceutical' adj2 service$') or 'pharmaceutical care' or pharmacies* or pharmacy* or pharmacist*).ti,ab. | 8237 |
| 3 | 1 AND 3 | 127 |

**Search Cochrane Library**

| **#** | **String** | **Results**  **(October 12, 2023)** |
| --- | --- | --- |
| 1 | [mh "chronic pain"] OR ((chronic:ti,ab OR persistent:ti,ab OR relentless:ti,ab OR enduring:ti,ab OR constant:ti,ab OR sustained:ti,ab OR continuous:ti,ab OR lingering:ti,ab OR protracted:ti,ab OR life-long:ti,ab OR continual:ti,ab OR continuing:ti,ab OR recurrent:ti,ab OR recurring:ti,ab) NEAR/4 (pain:ti,ab OR ache*:ti,ab OR cramp*:ti,ab OR spasm*:ti,ab OR colic*:ti,ab OR sore*:ti,ab OR tender*:ti,ab OR burn*:ti,ab OR arthralgia:ti,ab OR headache:ti,ab OR earache:ti,ab OR migraine:ti,ab OR mastodynia:ti,ab OR allodynia:ti,ab OR neuralgia:ti,ab OR hyperalgesia:ti,ab OR '*myalgia':ti,ab)) | 28949 |
| 2 | [mh Pharmacists] OR [mh "Pharmacy Service, Hospital"] OR [mh "Community Pharmacy Services"] OR [mh Pharmacy] OR [mh "Evidence-Based Pharmacy Practice"] OR [mh "Pharmaceutical Services"] OR (pharmaceutical NEAR/2 service):ti,ab OR "pharmaceutical care":ti,ab OR pharmacies*:ti,ab OR pharmacy*:ti,ab OR pharmacist*:ti,ab | 12132 |
| 3 | 1 AND 2 | 188 |
