# Supplementary material for: Characterising pharmacists’ interventions in chronic non-cancer pain care: a scoping review
Source: Int J Clin Pharm. 2024 Jun 11;46(5):1010–23. doi: 10.1007/s11096-024-01741-x (PMC11399199; doi:10.1007/s11096-024-01741-x)
Supplement: Supplementary file 2 — Supplementary file2 (DOCX 77 KB) [file 11096_2024_1741_MOESM2_ESM.docx]

## Supplementary file 3

**Characterising Pharmacists’ Interventions in Chronic Non-Cancer Pain Care: A Scoping Review**

**International Journal of Clinical Pharmacy**

Aljoscha Goetschi^a,b,*^, Carla Meyer-Massetti^a,c^

^a^Clinical Pharmacology and Toxicology, General Internal Medicine, University Hospital of Bern, Bern, Switzerland

^b^Graduate School of Health Sciences, University of Bern, Bern, Switzerland

^c^Institute for Primary Health Care (BIHAM), University of Bern, Bern, Switzerland

*corresponding author: aljoscha.goetschi@insel.ch

**Supplementary table 2**. This table shows the charted outcomes reported by each study. Green areas indicate improvement, blue indicates partial improvement, no colour implies no change, and red indicates negative outcomes.

|  | | | | | | | | | |
| --- | --- | --- | --- | --- | --- | --- | --- | --- | --- |
| **Study** | | **Outcomes** | | | | | | | |
|  |  | **Interventions on Methods, Measurement and Pain Assessment in Clinical Trials’ core outcome domains** | | | | | | **Other domain** | **Summary**  **Positive outcomes/**  **Total outcomes reported^1^** |
|  |  | **Pain intensity** | **Physical function** | **Emotional well-being** | **Satisfaction** | **Symptoms & ADEs** | **Disposition** | **Costs** |  |
| Alibaud R. *et al*.  2016  France (27) | | N | N | N | 100% (25/25) of patients were satisfied | 52% (13/25) of patients had ADEs^2^, 36% (9/25) had therapy optimisation, 36% (9/25) had non-pharmacological therapy in the beginning and 80% had it (20/25) at the end | N | N | 2/2 |
| Barrachina J. *et al*.  2022  Spain (28) | | N | N | N | N | 4 ADEs^2^ detected among 69 patients and treatment stopped. | N | N | 1/1 |
| Bauters T. *et al*.  2008  Belgium (29) | | N | N | N | N | 120 recorded interventions: 107 clinical interventions, 12 drug counselling sessions, 1 delivery of specific product. 95.3% (114/120) of clinical interventions were accepted. | N | N | 1/1 |
| Bellnier T. *et al*.  2022  USA (30) | | N | N | N | 89% (118/133) patients retained.  Anecdotally, patients seemed satisfied. | N | N | Revenues generated covered the time spent for all providers, plus some overheads. | 2/2 |
| Bhimji H. *et al*.  2020  Canada (31) | | N | N | N | N | Mean MME^3^ reduced from 129.8 to 108.2 mg/day. No change in adjunctive pain medication. 42.9% (6/14) of interventions were accepted. | N | N | 0.5/1 |
| Boren L. *et al*.  2019  USA (32) | | N | N | N | N | Pharmacists initiated non-opioid medications in 19.5% (209/1072) patient consultations.  Pharmacists screened more urine and signed agreements, but no significant overall change in MME^3^. Was a clinically significant mean reduction in MME^3^ after 5 pharmacist visits. Patients using physicians only had increased MME^3^. | N | N | 0.5/1 |
| Briggs M. *et al*.  2008  UK (33) | | Significant fall in mean pain score from 8/10 to 6.3/10. | N | N | In a subgroup, 92% (22/24) of patients were satisfied. | N | N | N | 2/2 |
| Bright D. *et al*.  2020  USA (34) | | N | N | N | N | Pharmacists triaged pharmacogenetic reports and recommended modifications. They also identified other non-pharmacogenetic-related problems and proposed changes. | N | N | 1/1 |
| Bruhn H. *et al*.  2013  UK (35)    Neilson A. *et al*.  2015  UK (62)^2^ | Remote MR | Lower chronic pain grades. | No change in physical function. | No change in anxiety and depression | N | 60% of interventions accepted. | N | More expensive for quality-adjusted life years similar to controls. | 1/5 |
|  | Prescribing | Lower chronic pain grades. | No change in physical function. | Less anxiety and depression. | In a subgroup, 85% (39/46) of patients were satisfied. | N | N | More expensive for quality-adjusted life years similar to controls. | 2/5 |
| Chelminski P. *et al*.  2005  USA (36) | | Mean pain score improved from 6.5/10 to 5.5/10 | Mean disability score improved from 47/70 to 39.3/70. | Mean depression score improved from 24/60 to 18/60.  More patients on antidepressants (44.4% to 52.4%) | N | Mean MME^3^ increased from 72 to 91 mg. | N | N | 2/4 |
| Chen M. *et al*.  2020  Canada (37) | | No significant changes in pain. | No change in pain interference or quality of life. | No change in pain interference or quality of life. | N | Pharmacists proposed 40 interventions, of which 60% (24/40) were accepted, and 45% (18/40) were still implemented at 3 months. They recommended activities for patients 22 times. | N | N | 1/4 |
| Coffey C. *et al*.  2019  USA (38) | | Mean pain score reduced from 8.3/10 to 5.6/10. | N | N | Patients were satisfied with the service but not with pain levels. | Mean immediate-release MME^3^ decreased by 7.9%, and mean extended-release MME^3^ increased by 5.5%. 88% of interventions were implemented. | 76–94% of patients (36-44/47) correctly answered questions in a quiz. | N | 3.5/4 |
| Conley M. *et al*.  2022  USA (39) | | N | N | N | N | 90% of eligible patients were seen (19/21). Excluding outliers, mean reduction of 24.9 MME^3^.  Recommended non-opioid therapies such as NSAIDs^4^ or paracetamol.  Recommended non-pharmacological therapies such as physical therapy or behavioural health. | N | N | 1/1 |
| Cox N. *et al*.  2018  USA (40) | | No significant change in pain scores. | N | N | N | Mean 26 MME^3^ reduction.  Less concurrent benzodiazepine use with opioids.  Mean number of non-opioid analgesics prescribed per patient rose from 2.1 to 2.4.  Patients using laxatives rose from 13% to 49%. | N | N | 1/2 |
| Dawson K. *et al*.  2023  Canada (41) | | Mean pain score decreased from 5.26/10 to 3.36/10. | Mean physical function score increased from 5.97/10 to 7.91/10. | A score summarising pain, physical function, mood, social activities, sleep quality and side effects improved from 16.01/31 to 9.18/31. | 73% (39/53) of patients felt their health and well-being improved. | Pharmacists documented 153 DRPs, and all their interventions were accepted by physicians. | Pharmacists intervened to improve adherence with 24% (13/53) of patients. | N | 6/6 |
| DeBar L. *et al*.  2022  USA (42) | | Mean pain score improved by 0.434/10 compared to controls. | Mean physical disability score improved by 0.06/24 compared to controls. | Mean general enjoyment of life, general activity and sleep score improved by 0.434/10 compared to controls. | Intervention group’s patient satisfaction was stable but declined in controls. | No change in MME^3^ but lower benzodiazepine rates in the intervention group. | N | N | 2.5/5 |
| Dole E. *et al*.  2007  USA (43) | | Significant reduction of pain scores with more visits: e.g. mean reduction of 1.1/10 from first to second visit. | N | N | N | N | N | Cost savings (of USD 455,238 per year) and revenue generation (of USD 107,550). | 2/2 |
| Duvivier H. *et al*.  2015  USA (44) | | No significant change in pain. | 45% (12/27) of patients were able to perform more activities of daily living, 55% (15/27) were able to perform fewer. | N | N | Adjunctive and complementary therapies were used.  No change in MME^3^. | Patients were more adherent to their therapy. | N | 1.5/4 |
| Faley B. *et al*.  2021  USA (45) | | 28% (5/18) of patients improved,  17% (3/18) worsened and 56% (10/18) had no change in pain. | 39% (7/18) of patients improved, 17% (3/18) worsened and 44% (8/18) had no change in combined pain, general activity and enjoyment of life. | 39% (7/18) of patients improved, 17% (3/18) worsened and 44% (8/18) had no change in combined pain, general activity and enjoyment of life. | N | Mean 3.8 mg reduction in MME^3^.  Interventions unrelated to opioids mainly focused on naloxone and adjustment of co-analgesics. | N | N | 2/4 |
| Fong GR  1975  USA (46) | | N | Increase in up-time activity in all but one patient. | N | N | Reduction in medication use by all patients. | N | N | 2/2 |
| Gammaitoni A. *et al*.  2000  USA (47) | | No change in pain scores. | No change in physical function. | No change in mood. | No change in patient satisfaction with therapy or with physical function. | Intervention group had better access to medication and better relief from therapy. The health clinic accepted 94% (15/16) of recommendations. | N | N | 1/5 |
| Hadi M. *et al*.  2016  UK (48) | | Significant mean reductions in pain scores (7/10 to 6/10) and in worst pain (8/10 to 7.5/10).  13/79 patients had clinically relevant pain reductions of 10-20%. | No change in physical function. | No change in depression or anxiety. | Majority of patients were satisfied with service. | Made pharmacological and non-pharmacological optimisations and referred patients to other disciplines. | N | N | 3/5 |
| Hay E.  2006  UK (49) | | Mean pain reduction in intervention at 3 months (-1.18/10), but not at 6 or 12 months compared to controls. | No change in physical function. | N | N | Fewer NSAIDs^4^ and more simple analgesics used in the intervention group. | N | N | 1.5/3 |
| Hoffmann W. *et al*.  2008  Germany (50) | | No change in number and intensity of attacks compared to controls. | No change in physical function compared to controls. | Better mental health compared to controls. | Patients generally satisfied with the service. 49% (98/201) of the intervention group felt a change in their life due to the intervention. | N | No change in self-management of headaches compared to controls. | N | 2/5 |
| Jorgenson D. *et al*.  2023  Canada (51) | | N | Minimal mean improvement in general health (3.1/7). | N | N | MME^3^ was reduced by 41.7 mg (from 230.7 mg to 189.0 mg).  Minimal changes to non-opioid medications. | N | N | 1.5/2 |
| Joypaul S. *et al*.  2018  Australia (52) | | N | 100% (252) of patients could better perform household chores and physical exercises. 14.6% (37/252) indicated they could work part-time again. | Mean pain self-efficacy score increased from 23.1/60 to 35.3/60. | N | More guideline-concordant medications: reduction of paracetamol duplication and increase in sustained release paracetamol. | N | N | 3/3 |
| Keen A. *et al*.  2017  USA (53) | | N | N | N | N | N | Nurses’ knowledge improved significantly. | N | 1/1 |
| Kientz J. *et al*.  1983  USA (54) | | N | N | N | N | 76% (19/25) of patients reduced their medications, 16% (4/25) were unchanged and 8% (2/25) had more. | N | N | 1/1 |
| Kroner B. *et al*.  2008  USA (55) | | N | N | N | N | 47 medication conversions made, of which 24 (51%) were persistent conversions. | N | For persistent conversions, medication costs avoided were approx. USD 465 in year 1. | 1.5/2 |
| Lagisetty P. *et al*.  2020  USA (56) | | No change in pain scores. | No change in physical health. | No change in enjoyment of life. | N | 74% (34/46) of interventions were accepted by physicians. No change in MME^3^. | N | N | 0.5/4 |
| Maleki S. *et al*.  2023  Australia (57) | | N | N | N | N | 22 interventions were made and accepted. | N | N | 1/1 |
| Manzur V. *et al*.  2020  USA (58) | | N | N | N | N | 84% (19/23) of patients were recommended non-pharmacological options to improve pain, mood and sleep. | 95% of patients (22/23) were advised about adherence. | N | 2/2 |
| Mayfield K.  2021  Australia (59) | | N | N | N | 100% patient satisfaction. | Significant reduction in MME^3^ (86.2 mg to 73.4 mg).  4/5 GPs found recommendations clear and practical and valued them. | Significant improvements in patient knowledge. | N | 3/3 |
| McDermott *et al*.  2006  UK (60) | | No change in pain scores. | No change in physical function. | No change in mental health. | N | 192 interventions were made for 86% of patients (120/140), of which 77% (148/192) were executed. | N | N | 1/4 |
| Mínguez Martí *et al*.  2005  Spain (61) | | N | N | N | N | 75% of patients (113/150) were able to up-titrate their dose (MME^3^ 22.3 mg). 25% (37/150) discontinued, mainly due to opioid intolerance. | N | N | 1/1 |
| Norman J. *et al*.  2017  USA (63) | | N | N | N | N | N | N | N | 0/0 |
| Petkova V.  2009  Bulgaria (64) | | Decrease in pain severity compared to controls. | No changes in physical function. | No changes in enjoyment of life or mood. | Patients were satisfied with pharmacy services. | Fewer visits to physicians than the controls.  Fewer ADEs^2^ than controls. | More compliant with therapy than controls. | N | 4/6 |
| Read R. *et al*.  1998  UK (65) | | Among 14 patients with follow-up, 9 had improved pain. | N | N | N | Of 28 patients with poor outcomes, 24 had changes proposed to their physician. | N | N | 2/2 |
| Richet E. *et al*.  2022 France (66) | | Among patients with follow up, 75% (9/12) had better pain control. | Of those with improved pain, 78% (7/9) could perform daily activities better. | Of those with improved pain, 67% (6/9) slept better and 44% (4/9) had better morale. | Patients were satisfied with the service. | 74% (14/19) of patients reported at least one ADE^2^ with dronabinol;  68% (13/19) continued treatment for longer than 3 months; 32% (6/19) needed to discontinue due to ADEs^2^. | N | N | 4.5/5 |
| Rife T. *et al*.  2021  USA (67) | | N | N | N | N | Pharmacists often recommended non-benzodiazepine alternatives. Of these recommendations, 41% (50/121) were not approved, 23% (28/121) were short-term approved, and 5% (6/121) were approved. | N | N | 1/1 |
| Semerjian M. *et al*.  2018  USA (68) | | N | N | N | N | Mean number of interventions per patient consultation was 2, and an intervention took place in 98.7% of consultations (375/380). | N | N | 1/1 |
| Skomo M. *et al*.  2008  USA (69) | | N | N | N | N | Intervention had no effect on mean number of consultations with physicians. | N | N | 0/1 |
| Slipp M. *et al*.  2017  Canada (70) | | Mean pain intensity score decreased from 5.8/10 to 4.1/10. | Mean pain disability score decreased from 37.6/70 to 23.8/70. | Mean pain disability score decreased from 37.6/70 to 23.8/70. | Patients were satisfied with the service. | Relief from pain medication increased from 24% to 45%. | N | The pharmacist–physician combination used 13 times less money to achieve the same pain reduction, and 19 times less money to achieve the same reduction in disability as the physician working alone. | 6/6 |
| Takahashi N. *et al*.  2018  Japan (71) | | Mean pain scores improved. | Mean muscle endurance and physical fitness improved, but no change in walking ability or static flexibility. | Anxiety, depression and overall quality of life improved. | N | N | N | N | 2.5/3 |
| Tilli T. *et al*.  2019  Canada (72) | | N | N | N | N | 66% (23/35) of patients received a care plan. Physicians declined the rest. 75% (24/32) of recommendations were accepted. Intervention clinics had more active opioid tapers. The number of co-prescriptions did not change. | N | N | 1/1 |
| Uejima K.  2019  Japan (73) | | Patients had improved pain scores. | Patients had better motor function. | Patients had less anxiety, depression and insomnia. | N | N | Patients showed higher compliance. | N | 4/4 |
| Weidman-Evans E. *et al*.  2009  USA (74) | | N | N | N | N | 19% increase in cardiac monitoring was achieved with no changes made by clinics without pharmacists’ advice. | N | N | 1/1 |
| Total | | 15/24 | 9/22 | 10/18 | 11.5/14 | 34/40 | 8/9 | 4/6 | 88.5/132 |
| Per cent | | 62.5% | 40.9% | 55.6% | 82.1% | 85.0% | 88.9% | 66.7% | 67.0% |
| ^1^ For the summary, we awarded one point if the domain improved, 0.5 of a point if the domain partially improved, 0 if there was no change, and -1 point if the domain deteriorated.  ^2^ ADE = Adverse Drug Event  ^3^ MME = Morphine Milligram Equivalents  ^4^ NSAIDs = Non-steroidal anti-inflammatory drugs | | | | | | | | | |
